# Diuretic dose trajectories in dilated cardiomyopathy: prognostic implications

**DOI:** 10.1007/s00392-022-02126-8

**Published:** 2022-11-17

**Authors:** Vincenzo Nuzzi, Antonio Cannatà, Pierpaolo Pellicori, Paolo Manca, Davide Stolfo, Caterina Gregorio, Giulia Barbati, Daniel I. Bromage, Theresa McDonagh, John G. F. Cleland, Marco Merlo, Gianfranco Sinagra

**Affiliations:** 1 Cardiothoracovascular Department, Azienda Sanitaria Universitaria Integrata Giuliano Isontina (ASUGI), University of Trieste, Via Pietro Valdoni 7, 34100 Trieste, Italy; 2 Department of Cardiovascular Science, Faculty of Life Science and Medicine, King’s College London, London, UK; 3 Robertson Centre for Biostatistics, Institute of Health and Wellbeing, Glasgow Royal Infirmary, University of Glasgow, Glasgow, UK; 4 Division of Cardiology, Department of Medicine, Karolinska Institutet, Stockholm, Sweden; 5 Biostatistics Unit, University of Trieste, Trieste, Italy; 6 MOX, Department of Mathematics, Politecnico di Milano, Milan, Italy; 7 National Heart & Lung Institute, Imperial College, London, UK

**Keywords:** Loop diuretics, Dilated cardiomyopathy, Heart failure, Long-term trajectories, Prognostic associations

## Abstract

**Background:**

For patients with heart failure, prescription of loop diuretics (LD) and of higher doses are associated with an adverse prognosis. We investigated LD dose trajectories and their associations with outcomes in patients with dilated cardiomyopathy (DCM).

**Methods:**

Associations between outcomes and both furosemide-equivalent dose (FED) at enrolment and change in FED in the subsequent 24 months were evaluated. According to FED trajectory, patients were classified as (i) dose↑ (FED increase by ≥ 50% or newly initiated); (ii) dose↓ (FED decrease by ≥ 50%); (iii) stable dose (change in FED by < 50%); and (iv) never-users. The primary outcome was all-cause-death/heart transplantation/ventricular-assist-device/heart failure hospitalization. The secondary outcome was all-cause-death/heart transplantation/ventricular-assist-device.

**Results:**

Of 1,131 patients enrolled, 738 (65%) were prescribed LD at baseline. Baseline FED was independently associated with outcome (HR per 20 mg increase: 1.12 [95% CI 1.04–1.22], *p* = 0.003). Of the 908 with information on FED within 24 months from enrolment, 31% were never-users; 29% were dose↓; 26% were stable dose and 14% were dose↑. In adjusted models, compared to never-users, stable dose had a higher risk of the primary outcome (HR 2.42 [95% CI 1.19–4.93], *p* = 0.015), while dose↑ had the worst prognosis (HR 2.76 [95% CI 1.27–6.03], *p* = 0.011). Results were similar for the secondary outcome. Compared to patients who remained on LD, discontinuation of LD (143, 24%) was associated with an improved outcome (HR 0.43 [95% CI 0.28–0.65], *p* < 0.001).

**Conclusions:**

In patients with DCM, LD use and increasing FED are powerful markers of adverse outcomes. Patients who never receive LD have an excellent prognosis.

**Graphical abstract:**

**Figure:**
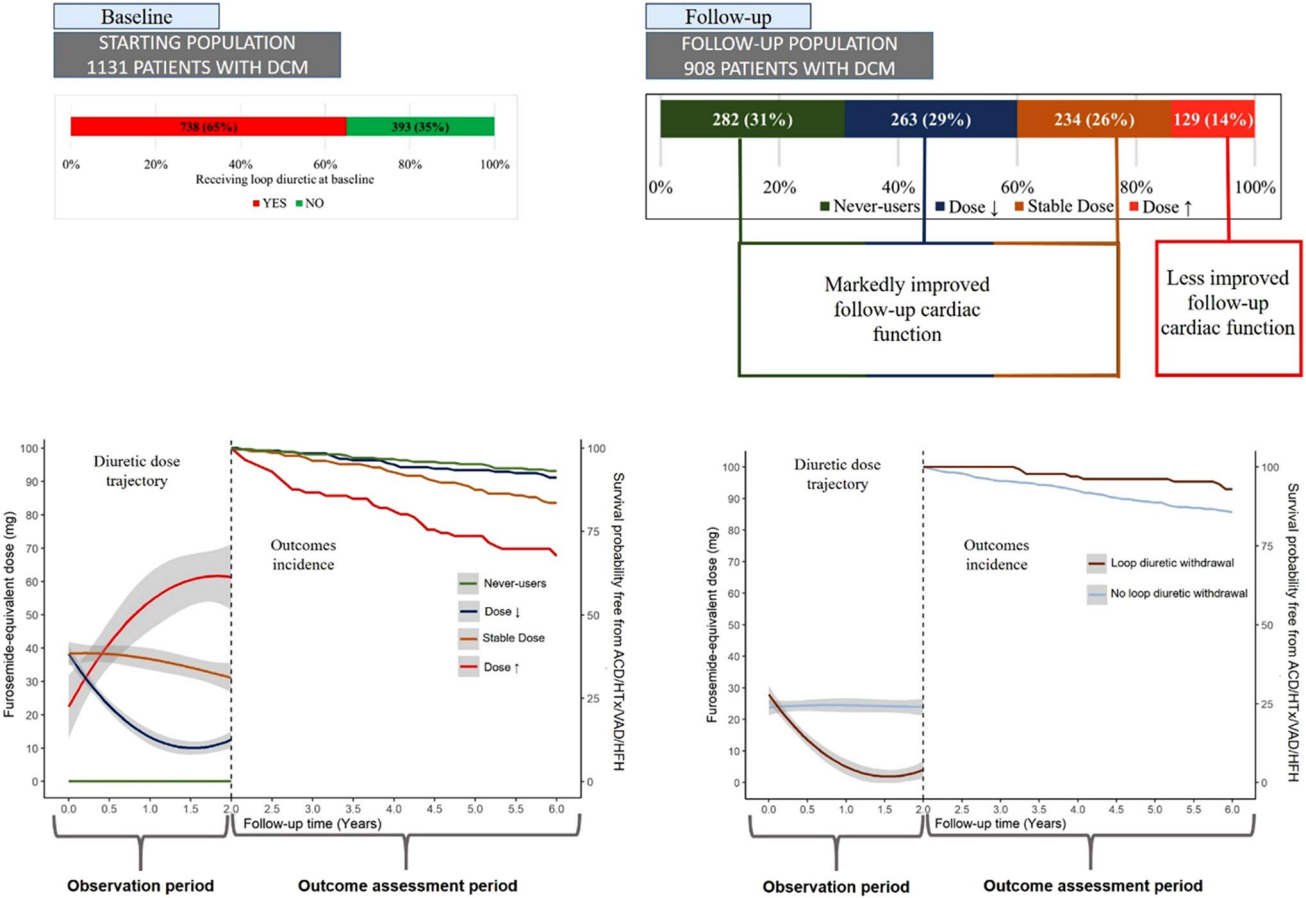
Among 1131 DCM patients 65% received loop diuretics at enrolment (upper left side). The bar chart on the upper right side shows the categorization in never-users/ dose↓/stable dose/ dose↑ over 24 months of follow-up. At the bottom is reported on the left side of each panel (observation period) the trajectory of LD dose in the four groups (left panel) and in patients who have their LD suspended vs those who continue LD (right panel) in the first two years. On the right side of each panel is shown the incidence of primary outcomes during the subsequent follow-up in the subgroups (outcome assessment)

**Supplementary Information:**

The online version contains supplementary material available at 10.1007/s00392-022-02126-8.

## Introduction

Loop diuretics (LD) are a cornerstone of treatment for heart failure (HF) to manage symptoms and signs of congestion, but their effect on long-term outcomes is controversial [1–3]. Observational studies suggest that higher doses of LD are associated with worse outcomes, perhaps because LD use and dose reflect the severity of congestion and cardiac dysfunction [4, 5]. However, LD might also accelerate disease progression by reducing the chance to up-titrate neurohormonal drugs, progressively worsen renal function and induce hypovolemia with consequent iatrogenic hypotension [6, 7].

So far, only a randomized trial including only 40 patients evaluated the effects of LD reduction in stable outpatients with chronic HF [8]. Current guidelines recommend using the lowest dose of LD to control signs and symptoms of congestion [1, 9], but the management of congestion is difficult [10]. Diuretic requirements may decline as disease-modifying therapies are introduced [11, 12] or might increase if the underlying disease progresses or complications, such as atrial fibrillation (AF), develop. In a sub-analysis of the CHAMPION trial, it has been proven that the most frequent therapy adjustment in chronic HF regards the LD dose, more frequently in terms of increased dose, suggesting that selected patients may require LD therapy intensification. Clinical experience and randomized trials suggest that discontinuing LD in carefully selected patients is feasible, safe and well tolerated [13]. On the other hand, suspending LD therapy can also cause a rapid and substantial increase in congestion in other patients [14]. Moreover, under-utilization of LD may lead to persistent congestion which might have adverse effects on cardiac remodelling and prognosis [9, 10].

Dilated cardiomyopathy (DCM) is a common cause of left ventricular ejection fraction (LVEF) impairment leading to HF with reduced (HFrEF) or mildly reduced LVEF. Recovery of LVEF is more common in patients with DCM than HFrEF due to other causes. [15]. LD therapy may no longer be required if cardiac function and congestion improve substantially, although the clinical course of DCM may be influenced by several other factors, such as the development of left bundle branch block (LBBB), AF and renal dysfunction and may also be truncated by sudden death [11]. Regular follow-up, with effective implementation of medical and device therapy, generally improves cardiac function and survival [11]. However, the trajectories of LD prescription in DCM were not previously explored and, therefore, the clinical associations and the prognostic implications of LD dose changes are still unknown. The longitudinal evolution of clinical and instrumental findings, alongside the LD dose prescription changes, might provide relevant insights into the pathophysiology linking diuretics and outcomes. In the present study, we investigated the long-term trajectories in the dose of LD in patients with DCM, and their associations with cardiac function and prognosis.

## Methods

### Study population

Consecutive outpatients with DCM, enrolled in the Trieste Heart Muscle Disease Registry between January 1st 1990 and March 7th 2019 who had a complete clinical and echocardiographic evaluation at baseline, including information on LD dose, were included in this study [16, 17]. Patients who had at least one additional clinical assessment performed within 24 (± 4) months after enrolment were considered for further analyses on the diuretic trajectory. Patients with significant coronary artery disease (> 50% stenosis of an epicardial coronary artery, ruled out by coronary angiography or computed tomography), a history of significant systemic hypertension (i.e. blood pressure > 140/90 mmHg), primary valve disease, active myocarditis, high alcohol intake (≥ 80 g/day [≥ 46 units per week], for at least 5 years), tachycardia-induced or peripartum cardiomyopathy, congenital heart disease or history of any advanced extra-cardiac disease (i.e. terminal cancer) associated with poor short-term prognosis were excluded [18].

Daily furosemide equivalent dose (FED) was calculated by multiplying doses of torasemide by 4 and doses of bumetanide by 40 [1, 19]. The FED was recorded at each visit, whenever possible. All beta-blockers and renin–angiotensin system inhibitors were converted into bisoprolol- and ramipril-equivalent doses, respectively (Supplementary Table S1).

Patients were classified according to the LD dose trajectory observed in the first 24 months of follow-up as follows: (i) dose↑, if LD dose was increased by at least 50% compared to baseline or the patient was initiated on an LD; (ii) dose↓, if LD dose decreased by at least 50% or if LD was withdrawn; (iii) never-users, if patients never received LD therapy; and (iv) stable dose, all remaining patients. For patients with multiple visits during the first 2 years, the last visit available before the 2 years of follow-up was considered to define the LD dose trajectory. Twelve patients went through both an increase and a decrease of at least 50% in LD dose within the first 24 months and were not included in the analysis on follow-up diuretic trajectory. The study was approved by the institutional ethical boards and complied with the Declaration of Helsinki. All the patients provided written informed consent.

### Echocardiography and genetic test

Echocardiograms were recorded on digital media storage at the institutions’ echocardiographic core laboratories and analysed offline by experienced echocardiographers, blinded to patient clinical information and outcomes, according to the latest available recommendations [20]. Details are reported in the supplementary text.

A subgroup of patients underwent next-generation sequencing, as previously described [20]. We classified patients in Titin-truncating variant-related DCM vs other forms of DCM, on the basis of the specific natural history of Titin-truncating variant-related DCMs [21, 22]. Additional details are provided as supplementary material.

### Study outcomes

The primary outcome was a composite of all-cause mortality, heart transplantation, durable ventricular-assist device implantation and HF hospitalization (ACD/HTx/VAD/HFH). The secondary outcome was a harder outcome including only all-cause mortality, heart transplantation and durable ventricular-assist device implantation (ACD/HTx/VAD). Information about outcomes was obtained from official reports and hospital discharge letters; for patients coming from other regions, information regarding outcomes were collected by direct contact with patients, their families or general practitioners, from the regional healthcare data warehouse and death registers. The trend of clinical and echocardiographic parameters in the four groups of patients divided according to the LD temporal trajectories was analysed in order to study the association between LD dose change and longitudinal functional parameters at follow-up (i.e. LVEF, left atrium dimension, New York Heart Association class, mitral regurgitation (MR), heart rate, beta-blockers and renin–angiotensin system inhibitors dose). No patient was lost to follow-up concerning outcomes. For this analysis, the last day of follow-up was the 7th of April 2021.

### Statistical analysis

**Fig. 1:**
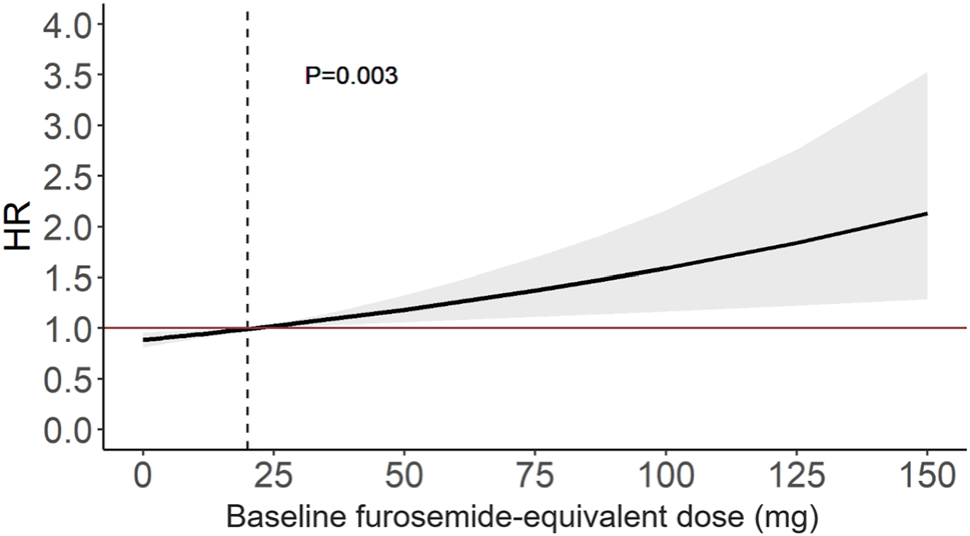
Association of furosemide-equivalent dose at enrolment and primary outcome. Reference value was set at a FED of 20 mg/day (vertical dotted line). HR for the primary outcome is adjusted for age, sex, enrolment period, systolic blood pressure, AF, NYHA III or IV, LVEF, LVEDVI, LAESD, moderate–severe MR, restrictive filling pattern, MRA and ICD implantation

Descriptive statistics are reported as mean and standard deviation, median and interquartile range [IQR], or counts and percentages, as appropriate. Comparisons of continuous variables were performed with cross-sectional comparisons between groups by the one-way ANOVA test, or the non-parametric Mann–Whitney *U*-test, when appropriate. The chi-square or Fisher exact tests were used for the comparisons of categorical variables. The trajectory of FED during the follow-up was illustrated using a smoothed conditional means analysis with penalized cubic regression splines. Most modifications of FED occurred within 24 months (Fig. 1) and consequently, the last available FED assessment in this period was used to define LD trajectory. Survival curves for the outcome measure were estimated using the Kaplan–Meier estimator and they were compared using the Log-Rank Test. Univariate and multivariable Cox regression models were fitted for the primary and secondary outcomes. For analyses of the relationship between baseline FED and outcomes, the baseline values for other variables were included in the multivariable models and the time was measured from enrolment. For analyses of the association between LD trajectories and outcomes the LD dose trajectory was treated as a time-depending variable; baseline was set at the 24th month of follow-up. Multivariable models were also taken from this assessment and the results were adjusted for variables recorded at 24 months visit. The same method was used for the assessment of the association between LD withdrawal and outcome. When there was evidence of non-linear effects between a continuous predictor and the primary outcome, the former was modelled using restricted cubic spline analyses with degrees of freedom. The variables considered in the multivariable model for adjustment were derived from univariate analysis. Variables with a statistical significance < 0.05 on univariate analysis were included in multivariable models. Baseline variables associated with diuretic withdrawal at 24-month follow-up were identified by univariate and multivariable Cox regression models. In the sensitivity analysis for the outcome, 19 patients with non-fatal events (i.e. HFH) that occurred between baseline and 2-year follow-up were excluded. The Cumulative Incidence Function was used to show the probability of LD withdrawal during follow-up, taking into account the competing risk of death. All statistical analyses were performed with IBM-SPSS (New York) version 25 and R statistical package version 3.6.2 (R Foundation, Vienna, Austria), with libraries “survival”, “ggplot2” and “splines”.

## Results

### Study population

Altogether 1156 patients with DCM were enrolled; of these, 25 did not have information on the use of LD at baseline and were excluded from the present analysis (consort diagram for this study is reported in Supplementary Fig. S1). This analysis included 1131 patients. At enrolment, 738 (65%) patients were receiving a LD and the median FED was 25 (IQR 25–50) mg/day; 508 (45%) received 1–40 mg of FED daily, 171 (15%) received 41–80 mg of FED/day and 59 (5%) received higher doses (Supplementary Table S2). Compared to never-users, those taking doses of LD > 80 mg/day were older, more likely to be men and to have AF and more severe symptoms (New York Heart Association class III/IV). They also had a lower LVEF, a larger left atrium (LA), were more likely to have a restrictive filling pattern (RFP) and were more likely to have moderate or severe MR.

### Prognostic role of baseline loop diuretic dose

Higher baseline FED, even after adjusting for potential confounders, was associated with the primary outcome in a linear fashion (HR per 20 mg increase: 1.12 [95% CI 1.04–1.22], *p* = 0.003) (Fig. 1).

### Follow-up evolution of loop diuretic prescription

**Fig. 2:**
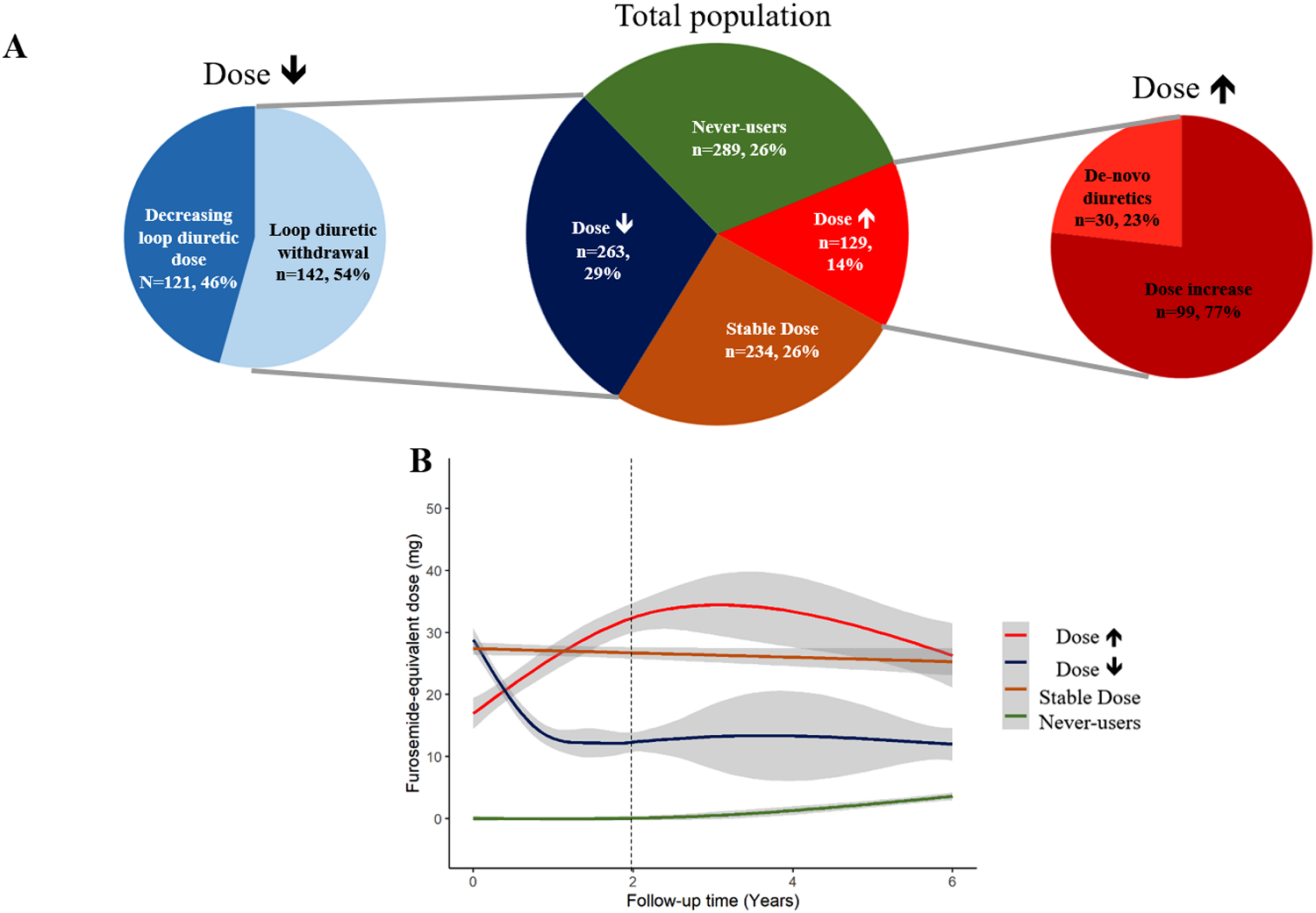
Changes, or lack of, in prescriptions of loop diuretics during the first 24 months of follow-up (908 patients) (**A**). Smoothed mean analysis showing the mean FED evolution during a long-term follow-up according to the trajectory in the first 24 months in the overall population (**B**). In green are represented never-users patients, in blue dose↓ patients, in brown stable dose patients and in red dose↑ patients; in grey are depicted the confidence intervals. The dotted lines represent the 24-month follow-up time

During the first 2 years of follow-up (23 [14–26] months), 908 (80%) patients had at least one other clinical assessment with information on LD dose. Among them, 129 (14%) patients were classified as dose↑, 234 (26%) as stable dose, 263 (29%) as dose↓ and 282 (31%) as never-users. Among dose↑, 99 (77%) had their LD dose increased by at least 50%, and 30 (23%) were newly initiated on LD therapy (Fig. 2A). Compared to other patients, never-users were younger, were less likely to have severe symptoms, AF or LBBB, had a higher LVEF, a smaller LA, and were less likely to have moderate–severe MR or an RFP (Table 1).

**Table 1:** Baseline characteristics of the study population with at least a second assessment of loop diuretic dose within 24 months with which to define diuretic trajectory (follow-up population)

908 patients	*N*	Dose↑ (129, 14%)	*N*	Stable dose (234, 26%)	*N*	Dose↓ (263, 29%)	*N*	Never-users (282, 31%)	*p*-value
Clinical evaluation									
Age, years	129	53 (39–63)	234	54 (46–63)	263	53 (44–62)	282	44 (34–56)	**0.002**
Men, no. (%)	129	87 (67)	234	157 (67)	263	186 (71)	282	204 (72)	0.547
Disease duration, months	120	7 (1–48)	215	5 (1–19)	243	3 (1–14)	228	12 (3–44)	** < 0.001**
Enrollment decade, no (%)									
1990–2000	129	48 (37)	234	66 (28)	263	91 (35)	282	102 (36)	0.507
2000–2010		43 (33)		85 (36)		92 (35)		90 (32)	
2010–2020		38 (30)		83 (36)		80 (30)		90 (32)	
Heart rate, bpm	122	71 (60–85)	224	74 (65–86)	251	75 (64–90)	274	69 (60–76)	0.063
AF, no. %	125	10 (8)	216	37 (17)	246	33 (13)	259	14 (5)	**0.004**
SBP, mmHg	125	120 (110–136)	231	120 (110–140)	257	120 (115–140)	275	120 (115–140)	0.298
NYHA III or IV, no. %	123	43 (35)	224	72 (32)	240	83 (35)	272	0 (0)	** < 0.001**
LBBB, no. %	126	54 (43)	232	85 (37)	260	70 (27)	278	69 (25)	** < 0.001**
Creatinine, mmol/l	112	95 (82–106)	203	90 (80–106)	244	91 (80–106)	209	88 (77–97)	**0.035**
Sodium, mEq/l	81	140 (138–142)	147	139 (138–142)	186	140 (138–141)	168	141 (138–142)	0.055
Genetic test									
TTNtv	34	6 (18)	67	13 (19)	70	18 (26)	101	15 (15)	0.360
Genetic negative/other variants	34	28 (82)	67	54 (81)	70	52 (74)	101	86 (85)	**–**
Echocardiography									
LVEF, %	129	29 (22–34)	234	28 (23–34)	263	28 (22–34)	282	38 (32–44)	**0.001**
LVEDVI, ml/m^2^	129	97 (78–123)	234	91 (78–117)	263	93 (76–118)	282	77 (68–93)	** < 0.001**
LAESD, mm	117	43 (38–48)	248	42 (37–48)	217	43 (38–49)	262	37 (32–42)	** < 0.001**
Moderate or severe MR, no. (%)	126	55 (44)	233	111 (50)	255	118 (46)	266	35 (13)	** < 0.001**
RFP, no. (%)	102	35 (34)	204	59 (29)	170	57 (34)	240	21 (9)	** < 0.001**
Medications and device therapy									
ACE-I or ARB or ARNI, no. (%)	129	128 (99)	234	232 (99)	263	262 (100)	282	257 (91)	** < 0.001**
Ramipril dose equivalent, mg	127	5 (2.5–10)	232	5 (2.5–10)	262	5 (3.75–10)	274	5 (2.5–7.3)	**0.031**
Beta-blockers, no. (%)	129	112 (87)	234	217 (93)	263	247 (94)	282	248 (88)	**0.026**
Bisoprolol dose equivalent, mg	99	2.5 (1.25–6.25)	197	2.5 (2.5–5)	228	3.75 (2.5–7.5)	233	3.75 (2.5–7.5)	0.080
MRA, no. (%)	129	63 (49)	234	153 (65)	263	164 (62)	282	30 (11)	** < 0.001**
Ivabradine, no. %	129	4 (3)	234	10 (4)	263	5 (2)	282	5 (2)	0.271
Diuretics, no. (%)	129	99 (77)	234	234 (100)	263	263 (100)	282	0 (0)	** < 0.001**
Furosemide dose equivalent, mg	129	25 (0–25)	234	25 (25–50)	263	25 (25–50)	282	0 (0–0)	** < 0.001**
CRT, no. %	129	24 (19)	234	48 (21)	263	27 (10)	282	27 (10)	** < 0.001**
ICD, no. %	129	48 (37)	234	81 (35)	263	73 (28)	282	71 (25)	**0.025**

*AF* atrial fibrillation, *SBP* systolic blood pressure, *NYHA* New York Heart Association, *LBBB* left bundle branch block, *TTNtv* Titin truncating variant, *LVEF* left ventricular ejection fraction, *LVEDVI* left ventricular end-diastolic volume index, *LAESD* left atrial end-systolic diameter, MR mitral regurgitation, *RFP* restrictive filling pattern, *ACE-i* angiotensin-converting enzyme–inhibitors, *ARB* angiotensin receptor blockers, *ARNI* angiotensin receptor neprilysin inhibitors, *MRA* mineralocorticoid receptors antagonists, *CRT* cardiac resynchronization therapy, *ICD* implantable cardioverter-defibrillator

*p*-values are estimated by the *χ*^2^ test for categorical variables; continuous variables are estimated by Student’s *t*-test

In bold are reported variables with significant differences among groups

### Loop diuretic dose trajectories, longitudinal echocardiographic assessment and genetic information

Substantial changes in FED were more frequent in the first 2 years of follow-up (Fig. 2B). On multivariable analysis, baseline factors associated with a reduction in FED were higher LVEF (HR per 1% 1.02 [95% CI 1.01–1.04], *p* = 0.006) and lower BMI (HR per kg/m^2^ 0.97 [95% CI 0.94–0.99], *p* = 0.022) (Supplementary Table S3).

**Fig. 3:**
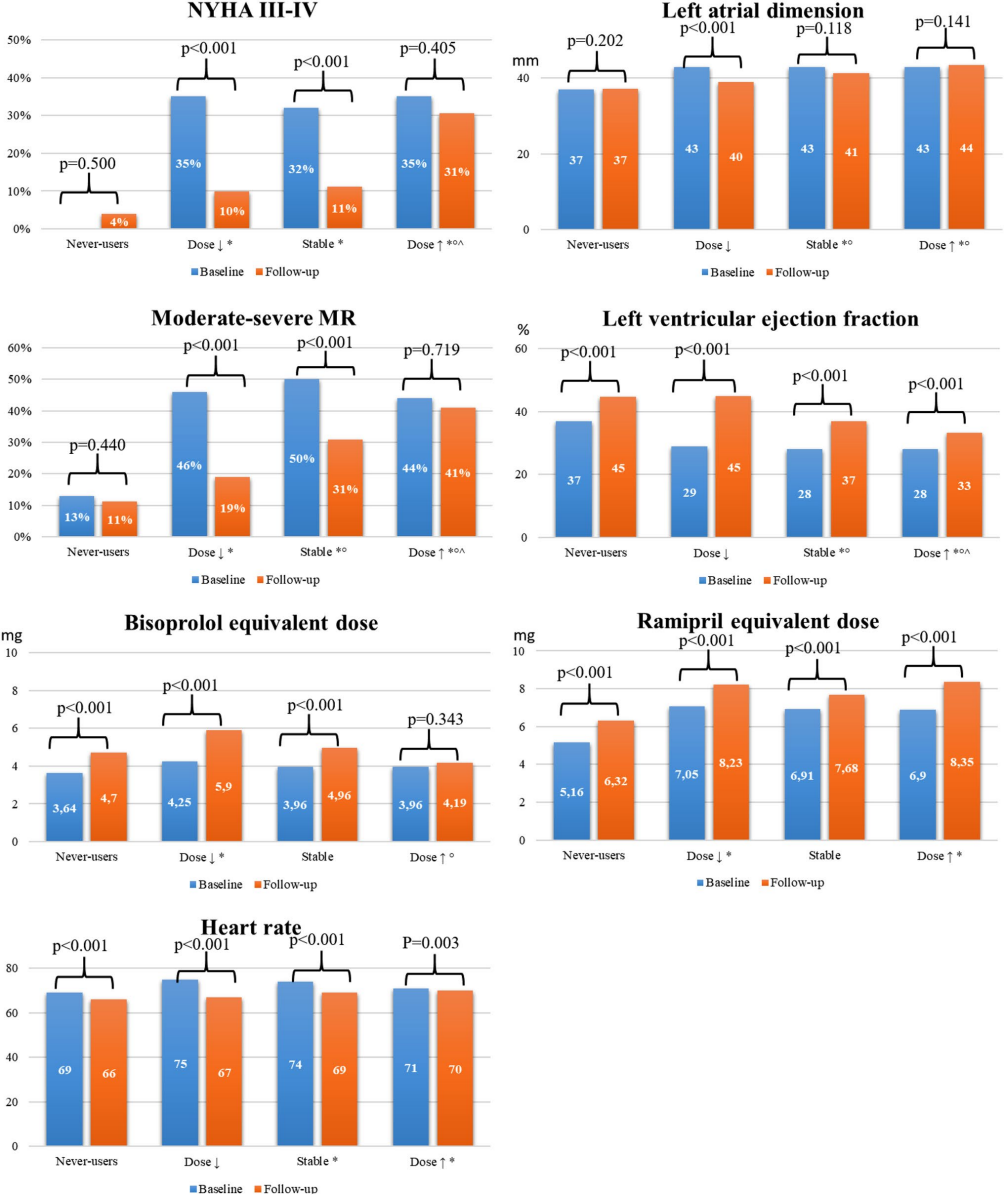
Baseline and 24-month follow-up assessment of the main echocardiographic characteristics, NYHA functional class and medical therapy in the four groups. 751 patients with available information on the diuretic trajectory and a paired echocardiographic at follow-up are included. *p*-values for the repeated measures are calculated with Mann–Whitney *U*-test. Ramipril equivalent dose includes the conversion of angiotensin converter enzyme inhibitors, angiotensin receptor antagonists and angiotensin neprilysin inhibitor. *p-value vs never-users < 0.05; °*p*-value vs dose < ↓0.05; ^*p*-value vs stable dose < 0.05

Of the 908 patients with at least two clinical assessments, 751 (83%) had an echocardiography paired with the LD dose reassessment. Substantial improvement in LVEF was observed for all patient subgroups; however, the proportion of patients who normalized their LVEF (e.g. LVEF ≥ 50%) during the first 2 years of follow-up was greater in dose↓ and never-users (27% and 23%, respectively) compared to dose↑ and stable dose (5% and 10%, respectively). Importantly, only dose↓ had a smaller LA compared to the initial echocardiographic assessment, which was associated with a reduction in moderate-severe MR (that declined from 35 to 10%). In contrast, the prevalence of moderate–severe MR at follow-up remained ≥ 40% for those who intensified LD therapy. Few patients classified as dose↓ or stable dose had severe symptoms on follow-up assessment; on the other hand, 41% of those with dose↑ remained severely symptomatic. Overall, doses of other treatments for HF increased during follow-up, except for beta-blockers among the dose↑, with a consequent higher heart rate compared to never users (Fig. 3).

Finally, we observed that, despite a similar dose of FED at enrolment for patients with and without TTNtv, the LD dose was reduced over the first 2 years of follow-up only for those with TTNtv (Supplementary Fig. S2).

### Association of loop diuretics trajectories with outcome

**Fig. 4:**
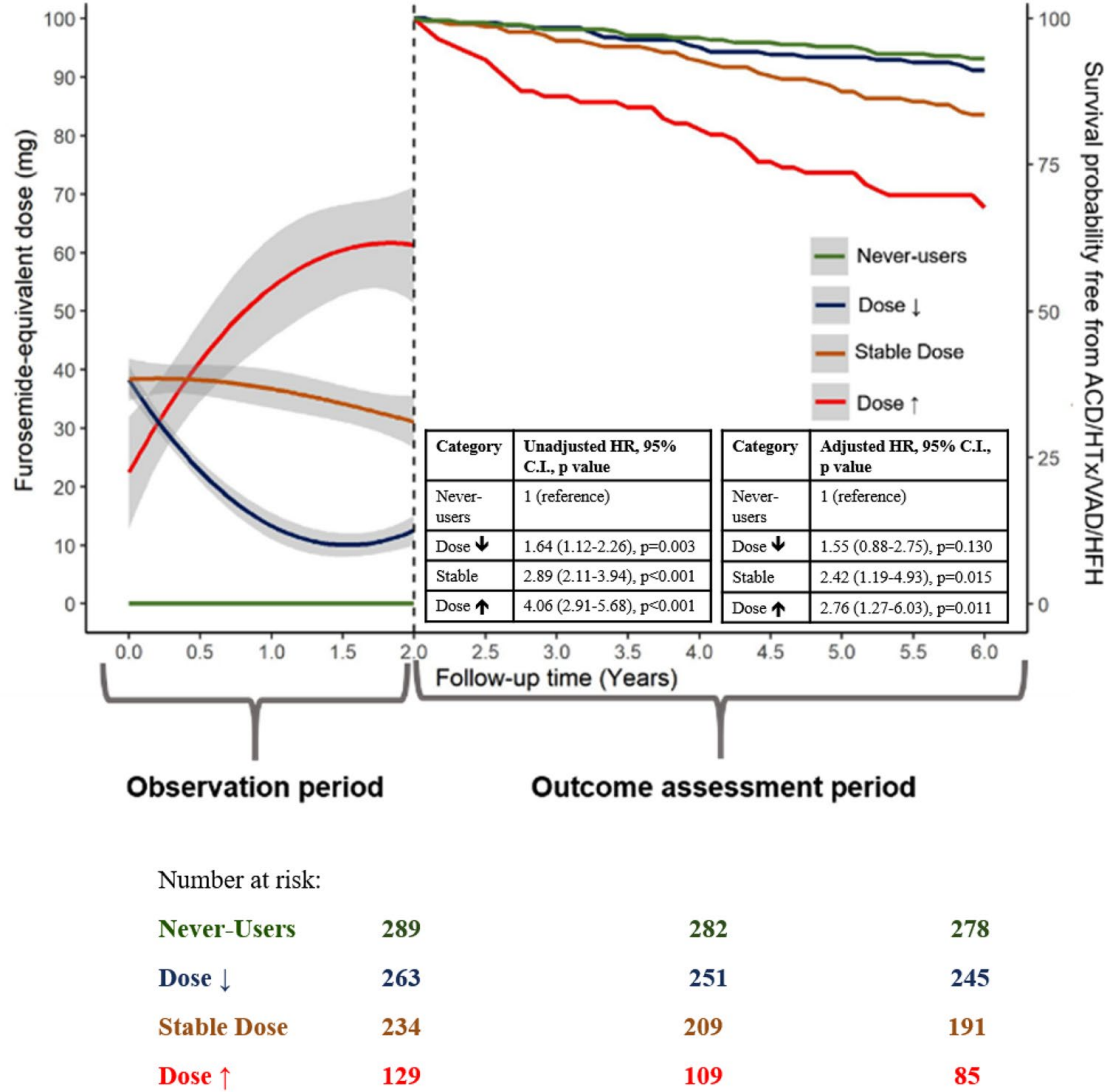
Kaplan–Meier analysis for the primary outcome according to the loop diuretics trajectory during the first 24 months. On the left side (observation period) is shown the trajectory of the FED in the four groups in the first 2 years. On the right side is shown the incidence of primary outcome during the subsequent follow-up in the subgroups (outcome assessment). HFH occurring before the follow-up visit was not considered. In green are represented never-users patients, in blue dose↓ patients, in brown stable dose patients and in red dose↑ patients. In the table are shown the unadjusted (left table) and adjusted (right table) HR, CI and *p*-values. Details regarding the multivariable model are reported in the text

Over a median follow-up of 122 (IQR 62–195) months, 412 (36%) patients met the study primary endpoint (248 deaths, 95 heart transplantations, 14 VAD, 115 HFH). Patients who had FED increased during follow-up had the worst outcome, while the risk was lowest for never-users (*p* < 0.001) (Fig. 4). The results were consistent setting the FED re-evaluation at 6- and 12-month follow-up (Supplementary Fig. S3). In adjusted models, compared to never-users patients (reference), stable dose patients had a more than the two-fold greater risk of ACD/HTx/VAD/HFH (HR 2.42 [95% CI 1.19–4.93], *p* = 0.015), while dose↑ had the poorest outcome (HR 2.76 [95% CI 1.27–6.03], *p* = 0.011) (Table 2). Similar results were observed for the secondary composite outcome (Supplementary Fig. S4). In sensitivity analysis, associations of LD trajectories with outcomes did not change when patients with non-fatal events (i.e. HFH, 20 patients) prior to the follow-up evaluation were excluded (Supplementary Fig. S5A, B).

**Table 2:** Time-depending univariate and multivariable analysis for primary outcome (ACD/HTx/VAD/HFH) considering the trajectory of diuretic dose over 24 months

	Univariate, HR (95% CI), *p*-value	Multivariable, HR (95% CI), *p*-value
Clinical evaluation		
Age, per year	1.020 (1.012–1.028), *p* < 0.001	1.003 (0.988–1.019), *p* = 0.670
BMI per Kg/m^2^	0.991 (0.966–1.016), *p* = 0.462	
Men	1.426 (1.111–1.832), *p* = 0.005	**1.950 (1.134–3.352), *p* = 0.016**
Disease duration per month	1.002 (0.998–1.005), *p* = 0.326	
Enrollment decade (compared to 1990–2000)	Reference	Reference
2000–2010	0.713 (0.547–0.929), *p* = 0.012	0.984 (0.518–1.871), *p* = 0.961
2010–2020	0.466 (0.306–0.710), *p* < 0.001	**0.429 (0.199–0.922), *p* = 0.030**
Heart rate per bpm	2.889 (1.939–4.303), *p* < 0.001	1.000 (0.977–1.025), *p* = 0.972
AF	1.825 (1.362–2.446), *p* < 0.001	1.287 (0.536–3.087), *p* = 0.572
SBP per mmHg	0.992 (0.984–0.999), *p* = 0.033	0.998 (0.987–1.009), *p* = 0.742
NYHA III or IV	4.387 (3.214–5.989), *p* < 0.001	1.136 (0.328–3.930), *p* = 0.840
LBBB	1.184 (0.943–1.488), *p* = 0.146	
Creatinine per mmol/l	1.001 (0.999–1.002), *p* = 0.222	
Diuretics trajectory (compared to never-users group)	Reference	Reference
Dose↓	1.639 (1.118–2.263), *p* = 0.003	1.554 (0.878–2.750), *p* = 0.130
Stable dose	2.885 (2.114–3.936), *p* < 0.001	**2.423 (1.190–4.932), *p* = 0.015**
Dose↑	4.060 (2.905–5.675), *p* < 0.001	**2.764 (1.266–6.031), *p* = 0.011**
Echocardiography		
LVEF per %	0.944 (0.933–0.955), *p* < 0.001	**0.971 (0.950–0.992), *p* = 0.008**
IVS per mm	0.980 (0.927–1.035), *p* = 0.468	
LAESD per mm	1.069 (1.051–1.087), *p* < 0.001	**1.045 (1.011–1.080), *p* = 0.008**
Moderate or severe MR	2.443 (1.861–3.208), *p* < 0.001	**2.254 (1.427–3.559), *p* < 0.001**
RFP	2.757 (1.858–4.091), *p* < 0.001	1.602 (0.872–2.944), *p* = 0.129
Medications and device therapy		
ACE-i/ARB/ARNI	1.185 (0.663–2.117), *p* = 0.567	
Ramipril/ARNI dose equivalent per mg	1.009 (0.989–1.028), *p* = 0.387	
Beta-blockers	0.986 (0.651–1.492), *p* = 0.945	
Bisoprolol dose equivalent per mg	0.978 (0.948–1.009), *p* = 0.163	
MRA	1.846 (1.422–2.396), *p* < 0.001	1.011 (0.564–1.810), *p* = 0.972
Ivabradine	1.874 (0.826–4.253), *p* = 0.133	
Furosemide dose equivalent per 20 mg	1.127 (1.106–1.150), *p* < 0.001	0.980 (0.871–1.103), *p* = 0.705
CRT	1.126 (0.838–1.513), *p* = 0.432	
ICD	1.290 (1.031–1.614), *p* = 0.026	**1**.045 (0.692–1.578), *p* = 0.833

All the variables are measured at 24 months

*BM* body mass index, *AF* atrial fibrillation, *SBP* systolic blood pressure, *NYHA* New York Heart Association, *LBBB* left bundle branch block, *LVEF* left ventricular ejection fraction, *IVS* interventricular septum, *LAESD* left atrial end-systolic diameter, *MR* mitral regurgitation, *RFP* restrictive filling pattern, *ACE-i* angiotensin-converting enzyme–inhibitors, *ARB* angiotensin receptor blockers, *ARNI* angiotensin receptor neprilysin inhibitors, *MRA* mineralocorticoid receptors antagonists, *CRT* cardiac resynchronization therapy, *ICD* implantable cardioverter-defibrillator

In bold are reported variables with significant association with the outcome at multivariable analysis

### Loop diuretics therapy withdrawal

Of the 608 patients who were taking LD at baseline, 143 (24%) had their LD stopped within 24 months; a further 49 (8%) patients had LD withdrawn during longer follow-up (Supplementary Fig. S6). Compared to those who had their LD withdrawn, those who continued LD were older, had a lower LVEF, a larger LA and were more likely to have moderate–severe MR, RFP and AF (Supplementary Table S4). On multivariable analysis, younger age, absence of moderate–severe MR and higher doses of beta-blockers were independently associated with stopping LD therapy within 24 months (Supplementary Table S5). LD therapy withdrawal within 24 months was associated with a reduced risk of the primary (HR 0.37 [95% CI 0.29–0.48], *p* < 0.001) and secondary (HR 0.34 [95% CI 0.26–0.45], *p* < 0.001) outcome (Supplementary Fig. S7).

## Discussion

In a large cohort of patients with DCM managed at a national tertiary-care center, use, higher daily dose and increasing the dose of LD all identified a greater risk of adverse outcomes. For patients who were not prescribed or discontinued LD, the prognosis was excellent (Graphic abstract). Changes in LD dose were more frequent during the 24 months and these modifications were associated with consistent changes in cardiac structure and function. For the subset of patients with genetic sequencing, only those with TTNtv-truncating variant-related DCM reduced their doses of diuretics during follow-up, perhaps suggesting a greater response to anti-HF therapy.

The principal therapeutic goals of managing DCM are prolonging life, control of symptoms, maintenance or improvement in quality of life and reduction in disability and morbidity. These goals can be achieved by implementing guideline-recommended pharmacological, device therapy, diuretics, and advices on life style [1, 18]. In our cohort, more than one-third of patients were not receiving LD at enrolment, which is perhaps a slightly higher proportion than other registries that enrolled outpatients with more diverse etiologies of HF [4, 11]. Several reasons may explain these differences. For instance, family screening programs for DCM patients might have allowed early diagnosis and initiation of HF treatment in our patients, when LV dysfunction was still asymptomatic; alternatively, a diagnosis of DCM may have been triggered by an arrhythmic event rather than symptoms or signs of congestion. Finally, patients with DCM might be characterized by a lower propensity to develop fluid overload compared to other forms of HF [23].

The association between LD use and higher doses with poor prognosis in ambulatory patients with HF is well known [4, 5], even among those with mild symptoms. In a post-hoc analysis of the EMPHASIS-HF trial, the use of LD was more strongly associated with prognosis than a history of HF hospitalization or plasma natriuretic peptide concentrations [24]. An association between the use of LD and adverse cardiovascular outcomes has been reported even in the absence of a diagnosis of HF for patients with type II diabetes and for those with AF. It is possible that the diagnosis of HF is often missed because signs and symptoms of congestion are masked by the use of LD [25].

The relationship between changes in LD treatment and prognosis in patients with HF has received less attention. Using the European Society of Cardiology Heart Failure Long-Term (ESC-HF-LT) Registry, Kapelios and colleagues showed that 16% of patients with all-etiologies HF had their LD dose increased and only 8% had it decreased; while in our DCM cohort, up to 29% of patients underwent LD down-titration [26]. They also found that LD dose escalation was associated with higher mortality but those with a reduction in LD dose had a similar outcome to those maintained on a stable dose. However, these authors used different criteria to define dose↓ or dose↑ (i.e. any change from baseline, respectively) and only included patients who were receiving an LD at enrolment.

The ReBIC-1 randomized trial demonstrated the feasibility of LD withdrawal in carefully selected outpatients with HFrEF, but did not provide information regarding long-term outcomes [13]. While neurohormonal antagonists should be continued for DCM lifelong to prevent clinical deterioration, [27], our findings suggest that when LD can be withdrawn without worsening congestion the subsequent risk of adverse cardiovascular events is low. Indeed, in this retrospective DCM population, when the treating physician considered it safe to reduce or even withdraw LD, the subsequent risk of adverse outcomes was significantly reduced. On the other hand, patients that may benefit from LD dose reduction have to be carefully selected, as in 40% of our cohort the LD dose was increased or maintained stable to avoid congestion and increased filling pressure.

The most likely explanation for the association between LD and prognosis is that patients with more severe cardiac and renal dysfunction also have more evidence of congestion, requiring treatment with LD to control symptoms and signs. Our results, showing the more favourable evolution of clinical and echocardiographic data in patients reducing their LD dose, support this hypothesis. Thus, LD are associated with a worse prognosis, which might be much worse for such patients if they were not used [5]. However, it is also possible that LD impair renal function and cause hypotension that reduces the ability to titrate other treatments for HF [6].

The analyses on the longitudinal trend of clinical and echocardiographic data according to the LD dose trajectories represent a novel result. We observed that patients who did not receive LD had better LV function and smaller ventricular and atrial volumes. Conversely, patients in whom LD were increased were less likely to show favourable cardiac reverse remodelling at follow-up. A dilated and dysfunctional LV will increase the severity of MR and LA dilation; eventually, this will lead to pulmonary and systemic venous hypertension and congestion requiring LD therapy. Further prospective studies are required to clarify if clinical and echocardiographic evolution might guide LD therapy in patients with DCM.

Interestingly, for the subset of patients with genetic characterization, reduction in LD appeared more common in those with TTNtv-related DCM, perhaps suggesting a better response to HF treatment [28]. This finding should be confirmed in other cohorts.

Currently, prognostic stratification for DCM is mostly based on the severity of LV systolic function on imaging, assessment of fibrosis by cardiac MRI, biomarkers, cardiopulmonary exercise testing or genetic studies [23, 29, 30]. Information on the use and dose of LD is readily available to healthcare providers. Our study demonstrates that LD use and dose are powerful prognostic markers of clinical outcomes; patients with DCM who do not take LD have an excellent prognosis and can be reassured. They should avoid interventions that are associated with risk or substantial costs and should be enrolled less in trials investigating the effects of new treatments on morbidity and mortality because there is little prospect that they will benefit but they might experience serious adverse effects. Patients prescribed with LD, especially at higher or increasing doses, are at much higher risk and require further investigation to identify remediable causes and should be included in randomized trials of new interventions. Altogether, these results support the notion that LD should be used as a pharmaco-epidemiological marker of underlying congestion, cardiac dysfunction, and adverse prognosis in patients with DCM.

### Limitations

The long enrolment period may have introduced a bias, as a treatment for HF has improved in the past few decades. However, in our centre, all patients with DMC and LV dysfunction received comprehensive treatment with anti-neurohormonal therapies since 1990 [31], and we adjusted for the enrolment period in our multivariable models. We report all-cause mortality; the incidence of non-cardiovascular death is low in younger patients with DCM [16]. We did not attempt to investigate possible differences between different types of LD because 99% of patients enrolled in this study were treated with furosemide. The FED change cut-off chosen to classify patients was defined according to the clinical significance of an increase of at least 50% in FED. Information on natriuretic peptides, genetic testing, cardiac magnetic resonance and clinical congestion was not systematically available. Finally, the retrospective nature allowed us to find prognostic associations as we could not exclude the presence of potential unmeasured confounders.

## Conclusion

For patients with DCM, LD use and increasing dose are associated with a poor prognosis and adverse cardiac remodelling. Conversely, in selected patients not receiving LD or being able to stop them is associated with a greater likelihood that ventricular function will recover and a good prognosis. LD use and dose should be included in prognostic models for DCM and considered as inclusion criteria in clinical trials when their objective is to reduce morbidity and mortality.

## Supplementary Information

Below is the link to the electronic supplementary material.

Supplementary file1 (DOCX 813 KB)

## Data Availability

All data are available upon reasonable request.
