# Correction: Diuretic dose trajectories in dilated cardiomyopathy: prognostic implications

**DOI:** 10.1007/s00392-022-02133-9

**Published:** 2022-12-06

**Authors:** Vincenzo Nuzzi, Antonio Cannatà, Pierpaolo Pellicori, Paolo Manca, Davide Stolfo, Caterina Gregorio, Giulia Barbati, Daniel I. Bromage, Theresa McDonagh, John G. F. Cleland, Marco Merlo, Gianfranco Sinagra

**Affiliations:** 1 Cardiothoracovascular Department, Azienda Sanitaria Universitaria Integrata Giuliano Isontina (ASUGI), University of Trieste, Via Pietro Valdoni 7, 34100 Trieste, Italy; 2 Department of Cardiovascular Science, Faculty of Life Science and Medicine, King’s College London, London, UK; 3 Robertson Centre for Biostatistics, Institute of Health and Wellbeing, Glasgow Royal Infirmary, University of Glasgow, Glasgow, UK; 4 Division of Cardiology, Department of Medicine, Karolinska Institutet, Stockholm, Sweden; 5 Biostatistics Unit, University of Trieste, Trieste, Italy; 6 MOX, Department of Mathematics, Politecnico di Milano, Milan, Italy; 7 National Heart & Lung Institute, Imperial College, London, UK

**Correction: Clinical Research in Cardiology** 10.1007/s00392-022-02126-8

Within the abstract, the following phrase in the ‘Methods’ section “According to FED trajectory, patients were classified as (i) dose (FED increase by ≥ 50% or newly initiated);” was corrected to read “According to FED trajectory, patients were classifed as (i) dose ↑ (FED increase by ≥ 50% or newly initiated);”. In the ‘Results’ section of the abstract, the sentence “Baseline FED was independently associated with outcome (HR per 20 mg increase: 1.12 [95% CI 1.04–1.22, *p* = 0.003].” was corrected to “Baseline FED was independently associated with outcome (HR per 20 mg increase: 1.12 [95% CI 1.04–1.22], *p* = 0.003).” Finally, in Table 1, the LVEF, % for Dose↓ patients was given incorrectly when it should have been “28 (22–34)” and the N value has been corrected from “263” to “282”. The original article has been corrected.

