# Supplementary material for: Diuretic dose trajectories in dilated cardiomyopathy: prognostic implications
Source: Clin Res Cardiol. 2022 Nov 17;112(3):419–30. doi: 10.1007/s00392-022-02126-8 (PMC9998319; doi:10.1007/s00392-022-02126-8)

**Supplementary text**

Left ventricular ejection fraction was calculated by Simpson's biplane method [1]. LV restrictive filling pattern was defined according to current guidelines [2]. Left atrium end systolic-diameter was measured in the parasternal long-axis view. Mitral regurgitation was assessed using a multiparametric approach and was categorized in mild vs moderate-severe [3].

With the use of next-generation sequencing (NGS), the genetic testing covered more than 95% of known DCM-related genes, as previously reported [4]. The variants were all validated with bidirectional Sanger sequencing and were therefore classified according to

current guidelines [5]. The minor allele frequency was verified in the gnomAD database (https://gnomad.broadinstitute.org/variant/22-46449891-G-A) and crosschecked with the ClinVar (http://www.ncbi.nlm.nih.gov/clinvar) and Cardio Classifier (https://www.cardioclassifier.org) databases. Rare variants in genes with similar functions or part of the same cellular compartment were clustered in different groups, as previously described [6].

**References**

1. Lang RM, Badano LP, Mor-Avi V, Afilalo J, Armstrong A, Ernande L et al. Recommendations for cardiac chamber quantification by echocardiography in adults: an update from the American Society of Echocardiography and the European Association of Cardiovascular Imaging. J Am Soc Echocardiogr. 2015 Jan;28(1):1-39.e14. doi: 10.1016/j.echo.2014.10.003.
2. Nagueh SF, Smiseth OA, Appleton CP, Byrd BF 3rd, Dokainish H, Edvardsen T et al. Recommendations for the Evaluation of Left Ventricular Diastolic Function by Echocardiography: An Update from the American Society of Echocardiography and the European Association of Cardiovascular Imaging. J Am Soc Echocardiogr. 2016 Apr;29(4):277-314.
3. Zoghbi WA, Adams D, Bonow RO, Enriquez-Sarano M, Foster E, Grayburn PA et al. Recommendations for Noninvasive Evaluation of Native Valvular Regurgitation: A Report from the American Society of Echocardiography Developed in Collaboration with the Society for Cardiovascular Magnetic Resonance. J Am Soc Echocardiogr. 2017;30(4):303-371. doi:10.1016/j.echo.2017.01.007.
4. Gigli M, Merlo M, Graw SL, Barbati G, Rowland TJ, Slavov DB et al. Genetic Risk of Arrhythmic Phenotypes in Patients With Dilated Cardiomyopathy. J Am Coll Cardiol. 2019 Sep 17;74(11):1480-1490. doi: 10.1016/j.jacc.2019.06.072.
5. Hershberger RE, Givertz MM, Ho CY, Judge DP, Kantor PF, McBride KL et al. Genetic Evaluation of Cardiomyopathy-A Heart Failure Society of America Practice Guideline. J Card Fail. 2018 May;24(5):281-302. doi: 10.1016/j.cardfail.2018.03.004. Epub 2018 Mar 19.
6. Dal Ferro M, Stolfo D, Altinier A, Gigli M, Perrieri M, Ramani F et al. Association between mutation status and left ventricular reverse remodelling in dilated cardiomyopathy. Heart. 2017 Nov;103(21):1704-1710. doi: 10.1136/heartjnl-2016-311017. Epub 2017 Apr 17.

Supplementary Table S1. Furosemide, bisoprolol and Ramipril conversions .

| **Furosemide dose equivalent** | Torasemide | Bumetanide |  |  |  |  |  |
| --- | --- | --- | --- | --- | --- | --- | --- |
|  | X 4 | X 40 |  |  |  |  |  |
| **Bisoprolol dose equivalent** | Carvedilol | Metoprolol | Atenolol |  |  |  |  |
|  | / 5 | / 20 | / 10 |  |  |  |  |
| **Ramipril dose equivalent** | Enalapril | Lisinopril | Captopril | Candesartan | Valsartan | Losartan | Sacubitril/valsartan |
|  | / 2 | /3.5 | / 15 | / 3.2 | / 32 | / 15 | 24/26 mg = 2.5  49/51 mg = 5 mg  97/103 mg = 10 mg |

| 1131 patients | N | Total population (1131, 100%) | No LDs (393, 35%) | FED daily dose 1-40 mg (508, 45%) | FED daily dose 40-80 mg (171, 15%) | FED daily dose >80 mg (59, 5%) | p-value |
| --- | --- | --- | --- | --- | --- | --- | --- |
| Age, years | 1131 | 50 (40-60) | 44 (34-56) | 53 (43-62) | 54 (43-63) | 51 (44-60) | 0.349 |
| Male sex, no. (%) | 1131 | 788 (70) | 277 (71) | 337 (66) | 123 (72) | 51 (86) | **<0.001** |
| Disease duration, months | 806 | 6 (1-27) | 9 (2-39) | 5 (1-23) | 4 (1-25) | 20 (2-80) | 0.103 |
| Enrollment decade  1990-2000  200-2010  2010-2019 | 1131 | 363 (32)  411 (36)  357 (32) | 137 (35)  129 (33)  127 (32) | 159 (31)  211 (42)  138 (27) | 50 (29)  54 (32)  67 (39) | 17 (29)  17 (29)  25 (42) | 0.008 |
| Heart rate, bpm | 1085 | 74 (17) | 69 (13) | 76 (18) | 79 (20) | 80 (16) | 0.062 |
| AF | 1033 | 117 (11) | 20 (6) | 58 (12) | 29 (18) | 10 (20) | **<0.001** |
| SBP, mmHg | 888 | 120 (110-140) | 120 (110-140) | 120 (110-140) | 120 (110-140) | 120 (110-130) | **0.047** |
| NYHA III or IV, no. % | 1072 | 235 (22) | 0 (0) | 132 (27) | 74 (47) | 30 (55) | **<0.001** |
| LBBB, no. % | 1114 | 343 (31) | 105 (27) | 167 (34) | 50 (30) | 21 (36) | 0.163 |
| TTNtv | 301 | 55 (18) | 18 (15) | 6 (12) | 29 (25) | 2 (18) | 0.360 |
| Genetic negative/other variant | 301 | 246 (82) | 106 (86) | 45 (88) | 86 (75) | 9 (82) | 0.097 |
| Creatinine, mmol/l | 914 | 89 (80-106) | 88 (76-97) | 90 (80-106) | 95 (80-107) | 97 (88-107) | 0.038 |
| Hb, g/dl | 852 | 14.1 (13.0-15.0) | 14.2 (13.3-15.2) | 14.1-13.2-14.9) | 13.9 (14.8-12.8) | 14.2-12.9-14.8) | 0.229 |
| Sodium, mEq/l | 684 | 140 (138-142) | 141 (139-142) | 140 (138-142) | 140 (138-142) | 138 (137-140) | **0.001** |
| LVEF, % | 1131 | 32 (25-39) | 38 (32-44) | 29 (24-35) | 28 (22-33) | 24 (20-30) | **<0.001** |
| LVEDVI, ml/m^2^ | 1131 | 87 (70-111) | 77 (64-96) | 89 (74-114) | 97 (81-123) | 105 (89-126) | **0.001** |
| LAESD, mm | 1042 | 41 (35-47) | 37 (32-42) | 42 (36-47) | 45 (40-50) | 51 (42-55) | **<0.001** |
| Moderate or severe MR, no. (%) | 1072 | 385 (34) | 56 (15) | 209 (43) | 84 (52) | 36 (66) | **<0.001** |
| RFP, no. (%) | 895 | 205 (18) | 33 (10) | 100 (25) | 55 (43) | 17 (46) | **<0.001** |
| ACE-I or ARB or ARNI, no. (%) | 1131 | 1091 (97) | 362 (92) | 505 (99) | 166 (97) | 58 (98) | **<0.001** |
| Ramipril dose equivalent, mg | 1118 | 5.0 (2.5-10.0) | 4.3 (2.5-7.5) | 5 (2.9-10) | 5 (2.5-10) | 5 (2.5-10) | 0.765 |
| Beta-blockers, no. (%) | 1131 | 1011 (89) | 336 (86) | 463 (91) | 158 (92) | 54 (92) | 0.020 |
| Bisoprolol dose equivalent, mg | 980 | 2.50 (1.25-5.0) | 2.5 (1.25-5) | 2.5 (1.9-5) | 2.5 (1.25-5) | 3.6 (1.25-5) | 0.696 |
| MRA, no. (%) | 1131 | 486 (43) | 39 (10) | 270 (53) | 127 (74) | 50 (85) | **<0.001** |
| Ivabradine, no. % | 1131 | 38 (3) | 5 (1) | 18 (4) | 8 (5) | 7 (12) | **<0.001** |
| Diuretics, no. (%) | 1131 | 738 (65) | 0 (0) | 508 (100) | 171 (100) | 59 (100) | - |
| Furosemide dose equivalent, mg | 1131 | 25.0 (0-25.0) | 0 (0-0) | 25 (12.5-25.0) | 50 (50-50) | 100 (100-125) | **<0.001** |
| CRT, no. % | 1131 | 140 (12) | 30 (8) | 69 (14) | 27 (16) | 14 (24) | **0.001** |
| ICD, no. % | 1131 | 304 (27) | 90 (23) | 144 (28) | 53 (31) | 17 (29) | 0.277 |

Supplementary Table S2. Characteristics of the total starting population divided according to the baseline diuretic dose.

AF, atrial fibrillation; SBP, systolic blood pressure; NYHA, New York Heart Association; LBBB, left bundle branch clock; LVEF, left ventricular ejection fractionLVEDVI, left ventricular end-diastolic volume index; LAESD, left atrial end-systolic diameter; IVS: interventricular septum; MR mitral regurgitation; RFP, restrictive filling pattern; ACE-i, angiotensin-converting enzyme–inhibitors; ARB, angiotensin receptor blockers; ARNI, angiotensin receptor neprilysin inhibitors; MRA, mineralocorticoid receptors antagonists; CRT, cardiac resynchronization therapy; ICD, implantable cardioverter-defibrillator. In bold are reported variables with significant differences amongst groups. Shadowed rows show variables with p value <0.001.

Supplementary Table S3. Univariate and multivariable analysis for dose🡻 diuretics trajectory. Only patients with available information regarding diuretic trajectory were included (follow-up population).

|  | Univariate, HR (95% C.I.), p value | Multivariable, HR (95% C.I.), p value |
| --- | --- | --- |
| Clinical evaluation | | |
| Age, per year | 0.992 (0.986-0.998), p=0.005 | 0.999 (0.992-1.007), p=0.876 |
| BMI per Kg/m^2^ | 0.960 (0.941-0.980), p<0.001 | **0.968 (0.941-0.995), p=0.022** |
| Male sex | 1.064 (0.882-1.282), p=0.518 |  |
| Disease duration per month | 1.000 (0.997-1.002), p=0.723 |  |
| Enrollment decade (compared to 1990-2000)  2000-2010  2010-2020 | 1.109 (0.904-1.361), p=0.320  1.213 (0.984-1.496), p=0.070 |  |
| Heart rate per bpm | 0.998 (0.993-1.003), p=0.393 |  |
| AF | 0.916 (0.678-1.238), p=0.569 |  |
| SBP per mmHg | 1.001 (0.997-1.006), p=0.583 |  |
| NYHA III or IV | 0.729 (0.583-0.911), p=0.005 | 0.997 (0.735-1.354), p=0.986 |
| LBBB | 0.717 (0.591-0.870), p=0.001 | 0.794 (0.615-1.024), p=0.076 |
| Creatinine per mg/dl | 1.001 (0.991-1.031), p=0.608 |  |
| Sodium per mEq/l | 0.995 (0.966-1.026), p=0.764 |  |
| Echocardiography | | |
| LVEF per % | 1.026 (1.017-1.036), p<0.001 | **1.021 (1.006-1.036), p=0.006** |
| LVEDD per mm | 0.972 (0.962-0.981), p<0.001 | 0.989 (0.974-1.004), p=0.989 |
| LAESD per mm | 0.980 (0.969-0.990), p<0.001 | 0.999 (0.982-1.016), p=0.900 |
| Moderate or severe MR | 0.720 (0.596-0.870), p=0.001 | 0.923 (0.705-1.208), p=0.559 |
| RFP | 0.721 (0.556-0.919), p=0.008 | 0.861 (0.641-1.155), p=0.318 |
| Medications and device therapy | | |
| ACE-i/ARB/ARNI | 0.730 (0.492-1.083), p=0.118 |  |
| Ramipril/ARNI dose equivalent per mg | 0.975 (0.958-0.992), p=0.005 | 0.986 (0.965-1.008), p=0.214 |
| Beta-blockers | 1.009 (0.754-1.350), p=0.953 |  |
| Bisoprolol dose equivalent per mg | 0.990 (0.964-1.016), p=0.444 |  |
| MRA | 0.794 (0.666-0.948), p=0.011 | 0.959 (0.743-1.239), p=0.750 |
| Ivabradine | 0.899 (0.480-1.685), p=0.741 |  |
| Furosemide dose equivalent per 20 mg | 0.970 (0.947-0.980), p<0.001 | 1.001 (0.996-1.007), p=0.689 |
| CRT | 1.535 (0.569-4.137), p=0.397 |  |
| ICD | 1.283 (0.764-2.153), p=0.346 |  |

BMI, body mass index; AF, atrial fibrillation; SBP, systolic blood pressure; NYHA, New York Heart Association; LBBB, left bundle branch clock; Hb, hemoglobin; LVEF, left ventricular ejection fraction; LVEDD, left ventricular end-diastolic diameter; LAESD, left atrial end-systolic diameter; MR mitral regurgitation; RFP, restrictive filling pattern; ACE-i, angiotensin-converting enzyme–inhibitors; ARB, angiotensin receptor blockers; ARNI, angiotensin receptor neprilysin inhibitors; MRA, mineralocorticoid receptors antagonists; CRT, cardiac resynchronization therapy; ICD, implantable cardioverter-defibrillator. In bold are reported variables with significant association at multivariable analysis

Supplementary Table S4. Comparison between patients withdrawing loop diuretics and patients not withdrawing loop diuretics.

| 608 patients | N | Loop diuretics withdrawal (143, 24%) | No loop diuretics withdrawal (465, 76%) | p-value |
| --- | --- | --- | --- | --- |
| Age, years | 608 | 48 (41-58) | 55 (46-64) | **<0.001** |
| Male sex, no. (%) | 608 | 95 (66) | 48 (34) | 0.313 |
| Disease duration, months | 576 | 3 (1-12) | 6 (1-29) | **0.007** |
| Enrollment decade  1990-2000  200-2010  2010-2019 | 608 | 59 (41)  46 (32)  38 (27) | 136 (29)  170 (37)  159 (34) | **0.024** |
| Heart rate, bpm | 595 | 76 (66-90) | 75 (64-88) | 0.204 |
| AF | 558 | 10 (8) | 70 (16) | **0.009** |
| SBP, mmHg | 595 | 123 (115-140) | 120 (110-140) | 0.858 |
| NYHA III or IV, no. % | 570 | 36 (27) | 162 (37) | **0.034** |
| LBBB, no. % | 599 | 38 (27) | 164 (36) | **0.031** |
| TTNtv | 162 | 13 (33) | 22 (18) | **0.047** |
| Creatinine, mmol/l | 535 | 90 (80-106) | 93 (80-106) | 0.553 |
| Hb, g/dl | 494 | 14.2 (13.2-15.1) | 14.0 (13.0-14.9) | 0.969 |
| Sodium, mEq/l | 395 | 140 (138-142) | 140 (138-142) | 0.900 |
| LVEF, % | 608 | 29 (23-37) | 28 (22-33) | 0.281 |
| LVEDVI, ml/m^2^ | 608 | 88 (74-119) | 93 (80-106) | 0.517 |
| LAESD, mm | 568 | 42 (37-47) | 43 (38-49) | 0.068 |
| Moderate or severe MR, no. (%) | 584 | 55 (40) | 226 (51) | **0.013** |
| RFP, no. (%) | 456 | 28 (23) | 123 (37) | **0.004** |
| ACE-I or ARB or ARNI, no. (%) | 608 | 143 (100) | 461 (99) | 0.341 |
| Ramipril dose equivalent, mg | 604 | 5.0 (2.5-10.0) | 5.0 (2.5-10.0) | 0.048 |
| Beta-blockers, no. (%) | 608 | 137 (96) | 426 (92) | 0.062 |
| Bisoprolol dose equivalent, mg | 511 | 3.75 (2.5-7.5) | 2.5 (1.25-5.0) | **0.021** |
| MRA, no. (%) | 608 | 75 (52) | 309 (67) | **0.002** |
| Ivabradine, no. % | 608 | 5 (4) | 23 (5) | 0.321 |
| Furosemide dose equivalent, mg | 608 | 25.0 (12.5-25.0) | 25.0 (25.0-50.0) | **0.001** |
| CRT, no. % | 608 | 13 (9) | 85 (18) | **0.005** |
| ICD, no. % | 608 | 41 (28) | 152 (33) | 0.213 |

BMI, body mass index; AF, atrial fibrillation; SBP, systolic blood pressure; NYHA, New York Heart Association; LBBB, left bundle branch clock; TTNtv: Titin truncating variant; Hb, hemoglobin; LVEF, left ventricular ejection fraction; LVEDD, left ventricular end-diastolic diameter; IVS, interventricular septum; LAESD, left atrial end-systolic diameter; MR mitral regurgitation; RFP, restrictive filling pattern; ACE-i, angiotensin-converting enzyme–inhibitors; ARB, angiotensin receptor blockers; ARNI, angiotensin receptor neprilysin inhibitors; MRA, mineralocorticoid receptors antagonists; CRT, cardiac resynchronization therapy; ICD, implantable cardioverter-defibrillator. In bold are reported variables with significant differences amongst groups.

Supplementary Table S5. Uni- and multivariable analysis for diuretic withdrawal within 24 months. Only patients taking diuretics at baseline with a second assessment within 24 months are included.

|  | Univariate, HR (95% C.I.), p value | Multivariable, HR (95% C.I.), p value |
| --- | --- | --- |
| Clinical evaluation | | |
| Age, per year | 0.978 (0.967-0.989), p<0.001 | **0.976 (0.954-0.997), p=0.029** |
| BMI per Kg/m^2^ | 0.951 (0.917-0.986), p=0.007 | 0.957 (0.893-1.025), p=0.211 |
| Male sex | 0.899 (0.636-1.272), p=0.549 |  |
| Disease duration per month | 0.991 (0.985-0.997), p=0.004 | 0.995 (0.986-1.004), p=0.283 |
| Enrollment decade (compared to 1990-2000)  2000-2010  2010-2020 | Reference  1.695 (1.127-2.548), p=0.011  1.109 (0.722-1.705), p=0.636 | Reference  1.276 (0.561-2.900), p=0.561 |
| Heart rate per bpm | 1.007 (0.998-1.016), p=0.118 | 0.589 (0.244-1.425), p=0.240 |
| AF | 0.916 (0.678-1.238), p=0.569 |  |
| SBP per mmHg | 1.010 (1.002-1.018), p=0.015 |  |
| NYHA III or IV | 0.677 (0.461-0.995), p=0.047 | 0.572 (0.264-1.242), p=0.158 |
| LBBB | 0.695 (0.479-1.008), p=0.055 |  |
| QRS duration per msec | 0.990 (0.984-0.996), p=0.002 | 0.994 (0.985-1.003), p=0.213 |
| Creatinine per mmol/l | 0.990 (0.565-1.449), p=0.677 |  |
| Sodium per mEq/l | 1.033 (0.974-1.097), p=0.276 |  |
| Echocardiography | | |
| LVEF per % | 1.022 (1.003-1.041), p=0.025 | 0.982 (0.943-1.002), p=0.369 |
| LVEDD per mm | 0.982 (0.964-1.001), p=0.062 |  |
| IVS per mm | 0.974 (0.910-1.042), p=0.437 |  |
| LAESD per mm | 0.975 (0.955-0.995), p=0.013 | 1.027 (0.977-1.079), p=0.298 |
| Moderate or severe MR | 0.663 (0.472-0.931), p=0.018 | **0.454 (0.219-0.939), p=0.033** |
| RFP | 0.557 (0.365-0.850), p=0.007 | 0.914 (0.418-2.000), p=0.823 |
| Medications and device therapy | | |
| ACE/ARB/ARNI | - |  |
| Ramipril dose equivalent per mg | 0.998 (0.966-1.031), p=0.891 |  |
| Beta-blockers | 1.958 (0.865-4.435), p=0.107 |  |
| Bisoprolol dose equivalent per mg | 1.063 (1.018-1.110), p=0.005 | **1.092 (1.027-1.161), p=0.005** |
| MRA | 0.597 (0.430-0.829),=0.002 | 0.905 (0.487-1.683), p=0.753 |
| Ivabradine | 0.680 (0.279-1.661), p=0.398 |  |
| Furosemide dose equivalent per 20 mg | 0.741 (0.633-0.867), p<0.001 | 0.787 (0.598-1.035), p=0.112 |
| CRT | 0.561 (0.079-4.012), p=0.565 |  |
| ICD | 0.692 (0.221-2.173), p=0.529 |  |

BMI, body mass index; AF, atrial fibrillation; SBP, systolic blood pressure; NYHA, New York Heart Association; LBBB, left bundle branch clock; Hb, hemoglobin; LVEF, left ventricular ejection fraction; LVEDD, left ventricular end-diastolic diameter; IVS, interventricular septum; LAESD, left atrial end-systolic diameter; MR mitral regurgitation; RFP, restrictive filling pattern; ACE-i, angiotensin-converting enzyme–inhibitors; ARB, angiotensin receptor blockers; ARNI, angiotensin receptor neprilysin inhibitors; MRA, mineralocorticoid receptors antagonists; CRT, cardiac resynchronization therapy; ICD, implantable cardioverter-defibrillator. In bold are reported variables with significant association with the event at multivariable analysis.

**Supplementary Figure S1.** Flow chart diagram of the study.


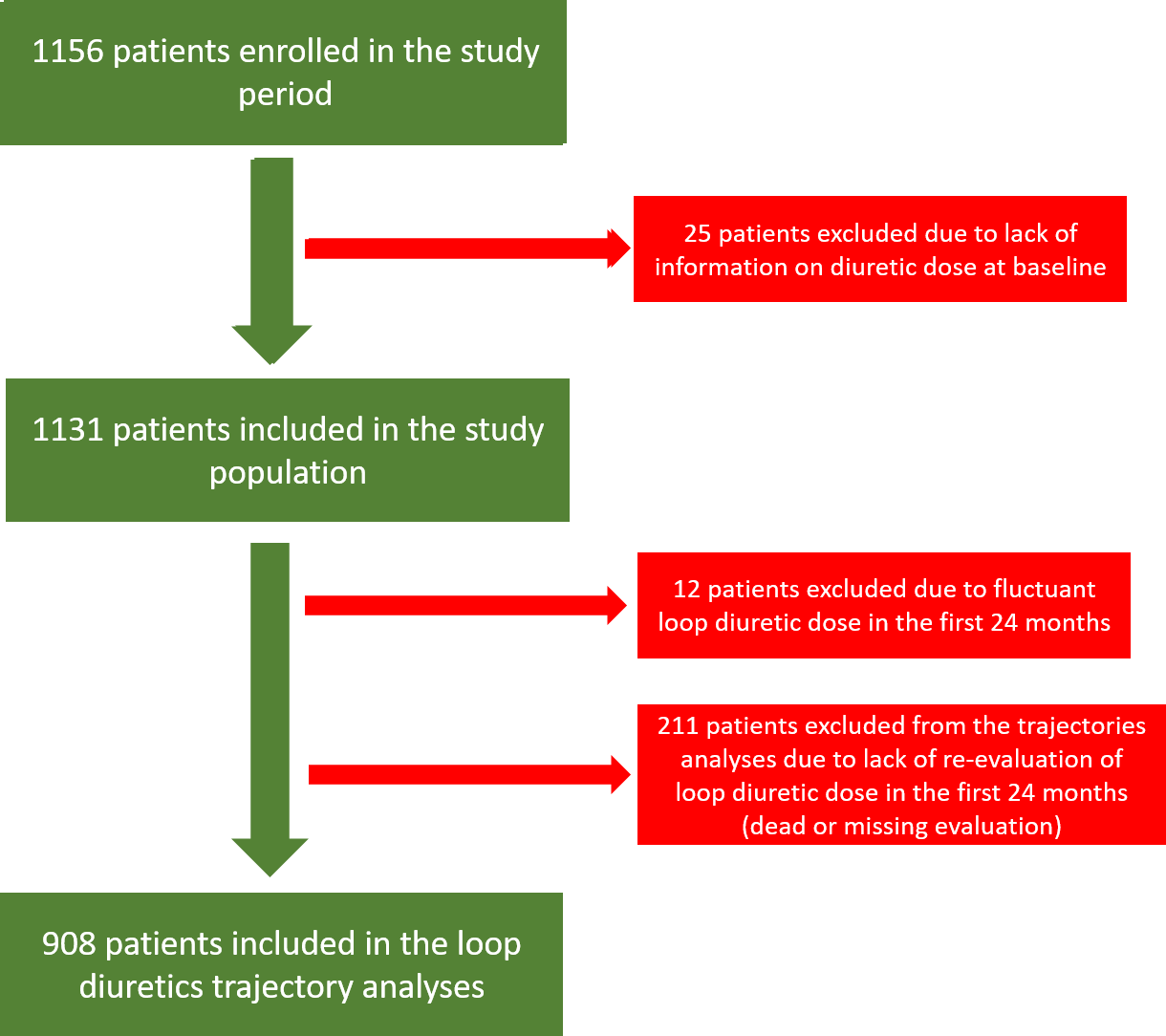


**Supplementary Figure S2.** Evolution of daily diuretic dose in TTNtv DCM vs non-TTNtv DCM.


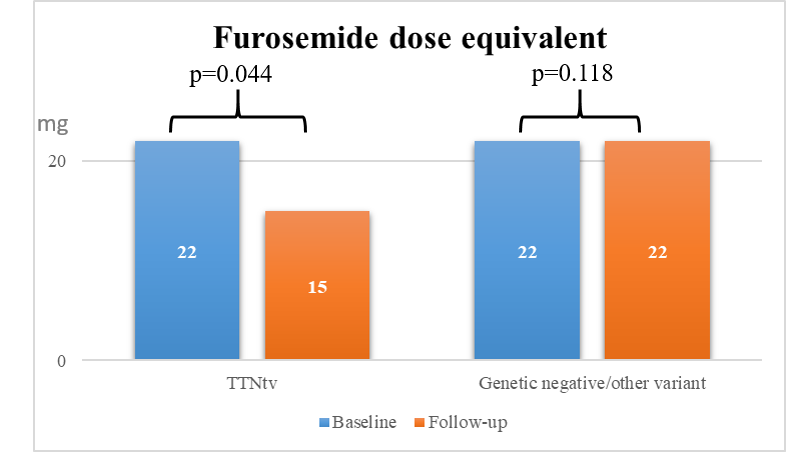


**Supplementary Figure S3.** Kaplan Meier analysis for the primary outcome according to the loop diuretics trajectory during the first 6 months (A) and 12 months (B) of follow-up. HFH occurring before the follow-up visit were not considered. In green are represented never-users patients, in blue dose🡻 patients, in brown stable dose patients and in red dose🡹 patients. In the table are shown the HR, C.I. and p-values.

**
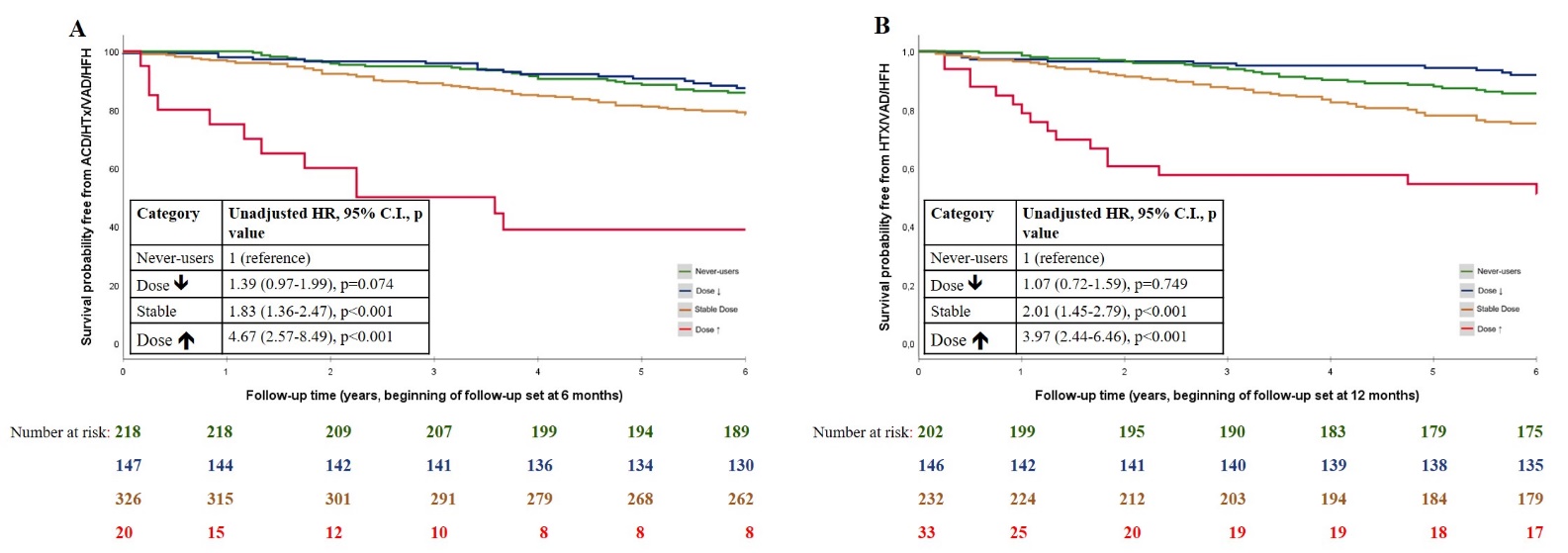
**

**Supplementary Figure S4**. Kaplan Meier analysis for secondary outcome according to the loop diuretics trajectory during the first 24 months. The beginning of the follow-up is set at 24 months (i.e. month 0 corresponds with the 24^th^ month after enrolment in the registry). HFH occurring before the follow-up visit were not considered. In green are represented never-users patients, in blue dose🡻 patients, in brown stable dose patients and in red dose🡹 patients. In the tables are shown the unadjusted (left table) and adjusted (right table) HR, C.I and p-values. Details regarding multivariable model are reported in the text.


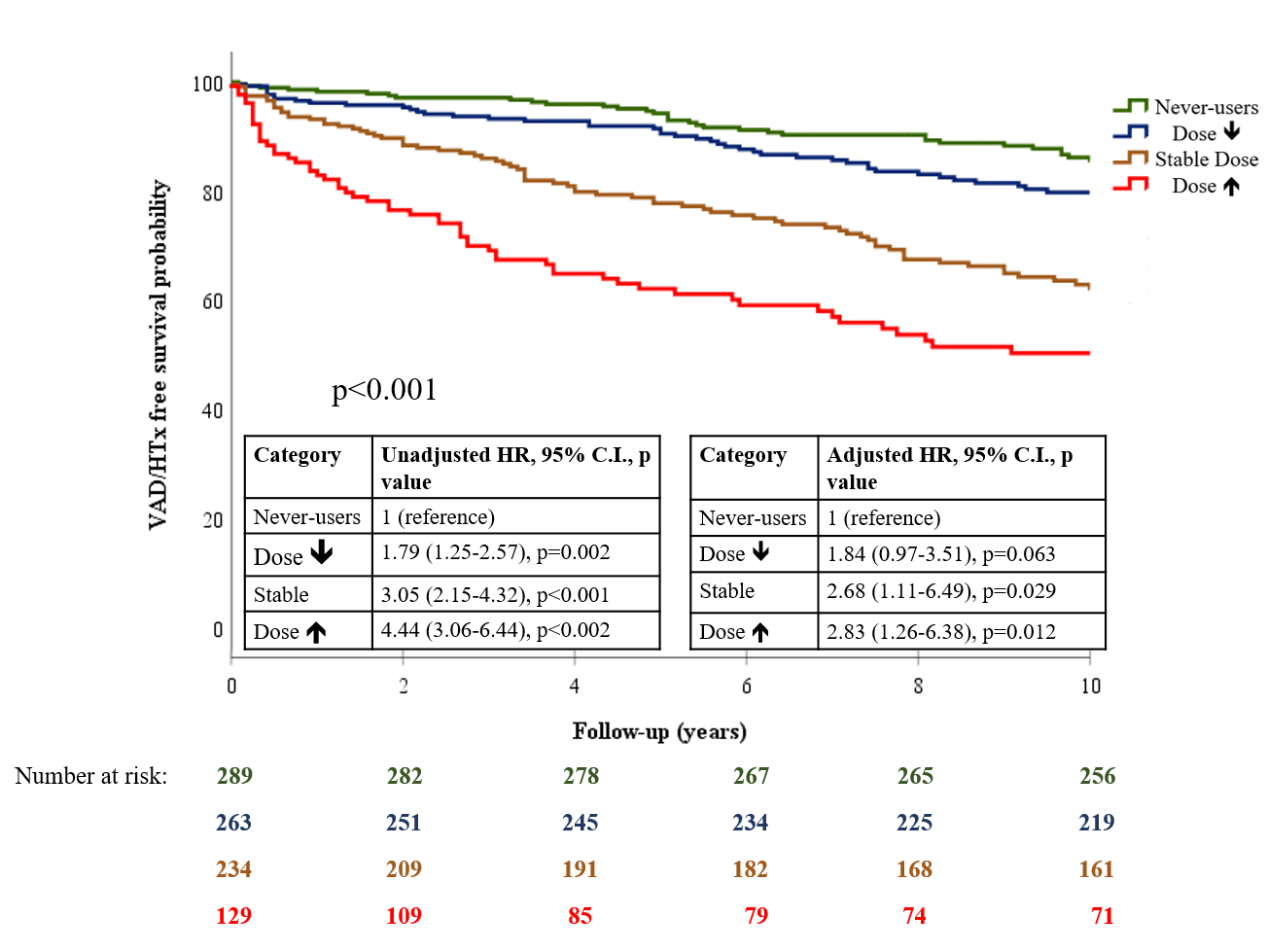


**Supplementary Figure S5.** Kaplan Meier sensitivity analysis for primary (A) and secondary (B) outcome according to the diuretics dose trajectory. Baseline is set at last evaluation within 24 months observation period (i.e. month 0 corresponds with the 24^th^ month after enrolment in the registry). Patients with heart failure hospitalization before the last evaluation before the 24^th^ month were excluded. In green are represented never-users patients, in blue dose🡻 patients, in brown stable dose patients and in red dose🡹 patients.


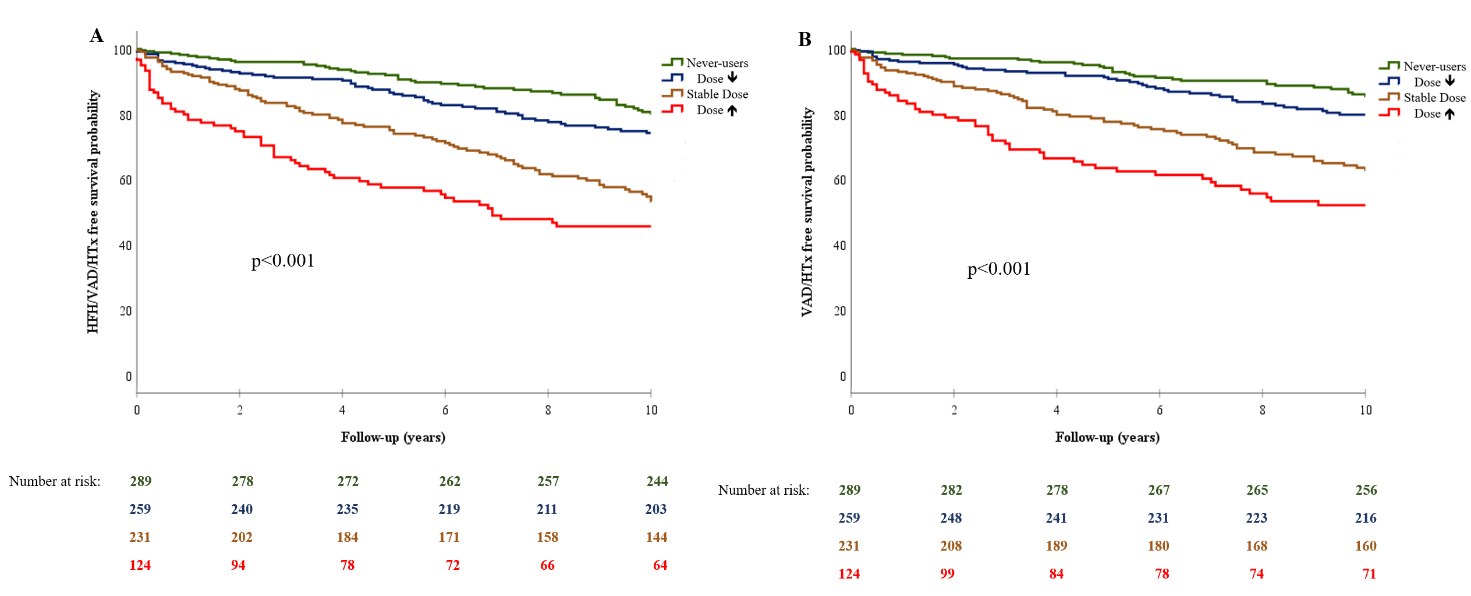


**Supplementary Figure S6.** Cumulative incidence function showing the incidence of diuretic withdrawal.**
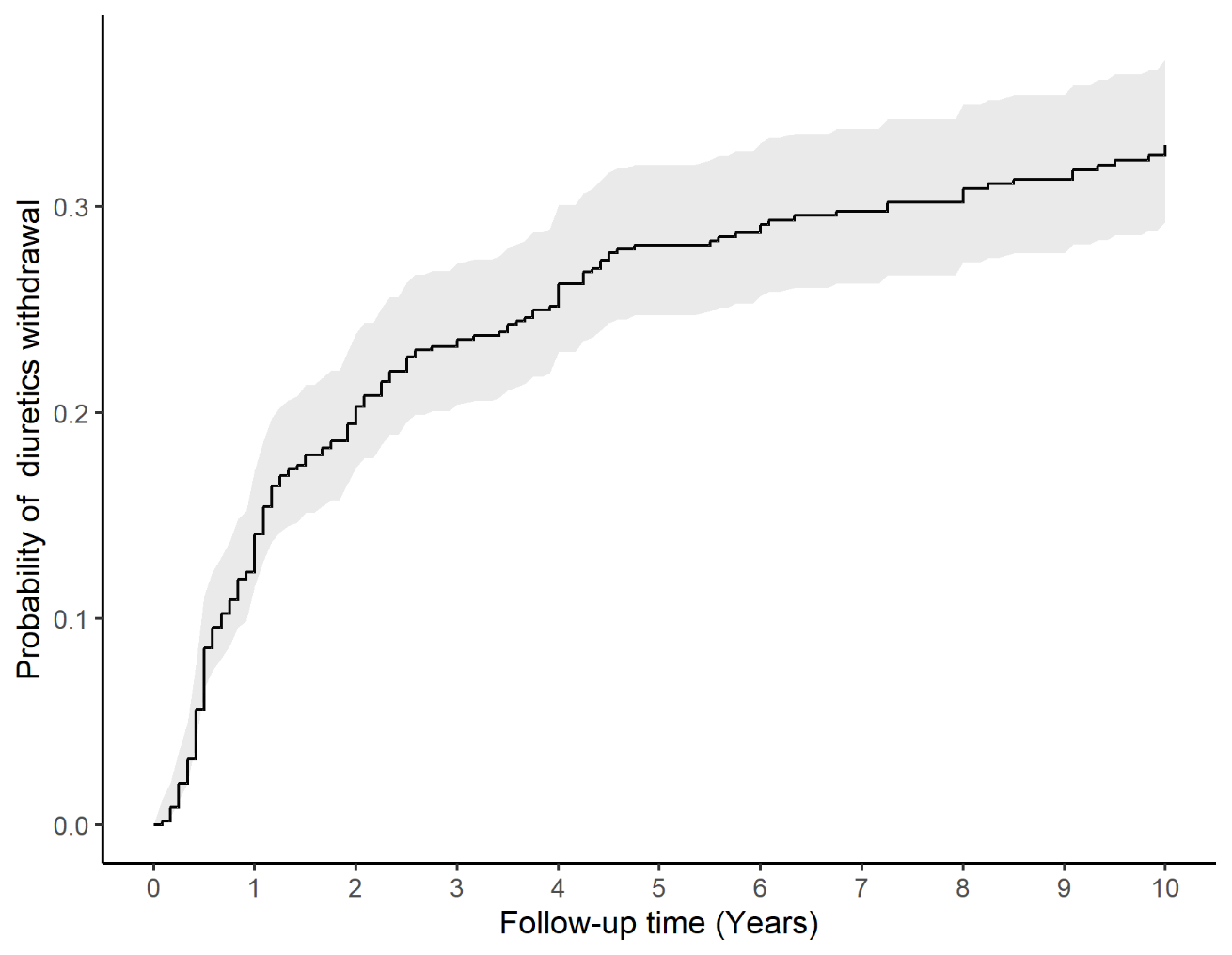
**

**Supplementary Figure S7.** Kaplan Meier analysis for primary (A) and secondary (B) outcome on population on LD at enrolment, divided according to LD withdrawal. The beginning of the follow-up is set on the 24 months follow-up visit (i.e. month 0 corresponds with the 24^th^ month after enrolment in the registry). HFH occurring within the first 24 months were not considered. In blue are represented patients undergoing diuretic withdrawal, in red are represented patients who did not stop their LD.


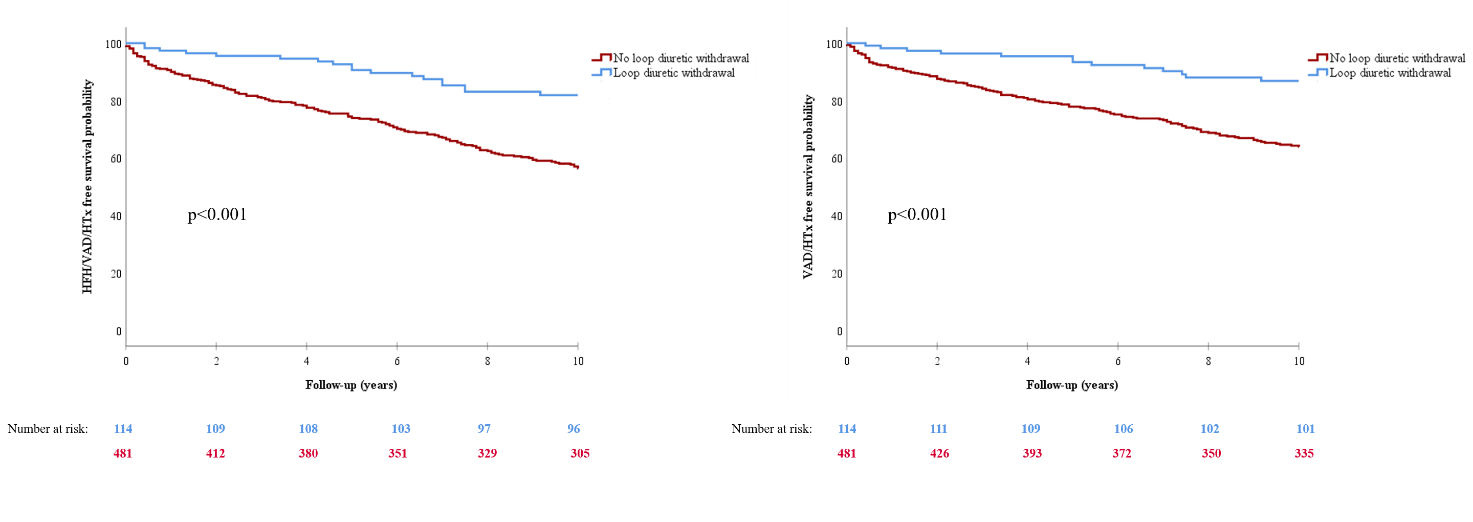

Supplement: Supplementary file 1 — Supplementary file1 (DOCX 813 KB) [file 392_2022_2126_MOESM1_ESM.docx]
